# Airway Management of Patient With Mounier–Kuhn Syndrome: A Case Report

**DOI:** 10.1002/rcr2.70429

**Published:** 2025-12-10

**Authors:** Ali Karami, Vida Naderi Boldaji, Fatemeh Khalili

**Affiliations:** ^1^ Anesthesiology and Critical Care Research Center Shiraz University of Medical Sciences Shiraz Iran

**Keywords:** airway management, anaesthesia, Mounier–Kuhn syndrome

## Abstract

Mounier–Kuhn syndrome (MKS) is a rare disorder characterised by tracheobronchomegaly, which poses significant anaesthetic challenges due to air leaks during mechanical ventilation. We report a 65‐year‐old male with previously undiagnosed MKS who underwent cardiac surgery. Following intubation with an 8‐mm endotracheal tube (ETT), a persistent air leak resulted in inadequate ventilation despite tube repositioning and upsizing to a 9‐mm ETT. Preoperative chest imaging revealed significant tracheal dilation. To achieve adequate ventilation, we implemented a modified technique: advancing the ETT into the right main bronchus, then retracting until bilateral breath sounds were audible, supplemented by gauze packing to reinforce the seal. This case underscores the importance of preoperative recognition of MKS to anticipate ventilation difficulties. The described technique, adapted from tracheoesophageal fistula management, effectively resolved the air leaks complication.

## Graphical abstract

Mounier–Kuhn syndrome (MKS) is a rare disorder characterised by tracheobronchomegaly, which poses significant anaesthetic challenges due to air leaks during mechanical ventilation. This report describes a 65‐year‐old male patient with MKS undergoing cardiac surgery and provides a comprehensive review of existing literature to enhance understanding and management strategies for this condition.

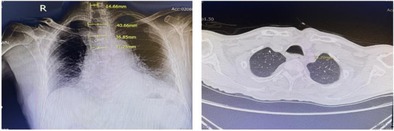

## 1 Introduction

Mounier–Kuhn syndrome (MKS) is a rare congenital condition characterised by significant enlargement of the trachea and main bronchi, resulting primarily from abnormalities in the elastic and muscular fibres of the tracheobronchial tree [1]. Patients with MKS present with a wide spectrum of clinical manifestations, ranging from asymptomatic status to recurrent respiratory infections that may progress to respiratory failure [2].

The anaesthetic management of patients with MKS poses particular challenges, chiefly due to the substantial risk of pulmonary aspiration and air leakage during mechanical ventilation. Anaesthesiologists must recognise these risks and prepare appropriate management strategies. Current literature on anaesthetic experiences with MKS remains limited, with only a few cases reported to date. This report describes a 65‐year‐old male patient with MKS undergoing cardiac surgery and provides a comprehensive review of existing literature to enhance understanding and management strategies for this condition.

## 2 Case Report

This study received approval from the Ethics Committee of Shiraz University of Medical Sciences (IR.SUMS.REC.1403.410). A 65‐year‐old male patient with hypertension, diabetes mellitus, and ischemic heart disease was scheduled for on‐pump coronary artery bypass grafting and redo aortic valve replacement. His medical history included a previous cerebrovascular accident resulting in right hemiplegia and aphasia. The patient reported no significant respiratory or infectious issues and no previous experience with general anaesthesia. Following arterial line insertion and standard monitoring induction of anaesthesia was achieved using midazolam (0.2 mg/kg), fentanyl (1.5 μg/kg), propofol (2 mg/kg), and cis atracurium (0.15 mg/kg). Intubation was performed with an 8‐mm internal diameter (ID) cuffed endotracheal tube (ETT), and the cuff was inflated after confirming bilateral lung sounds.

**FIGURE 1:**
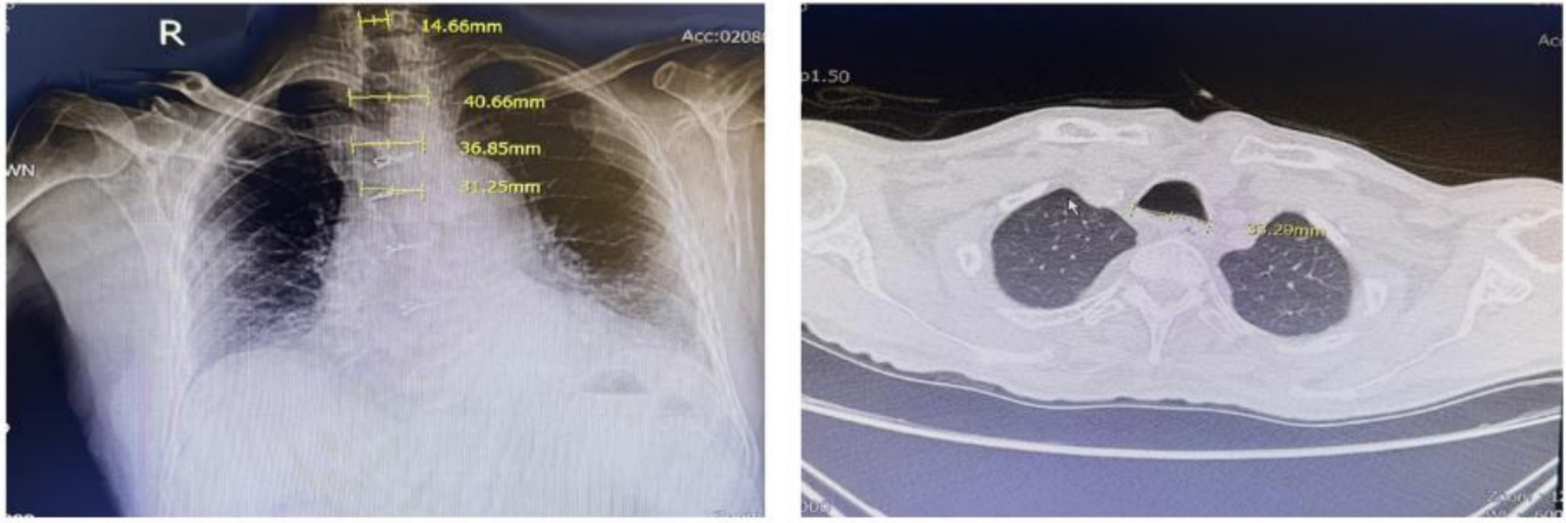
The preoperative chest x‐ray. The cross‐section chest CT image of the trachea, the trachea's maximum transverse diameter measured 33.29 mm.

Upon initiating mechanical ventilation, a significant air leak was detected. Despite correct ETT placement and appropriate tidal volume settings, air leakage persisted, resulting in inadequate tidal volume and insufficient minute ventilation. Manual ventilation was initiated, and the ETT was replaced with a 9‐mm ID tube with additional cuff inflation; however, the air leak continued. Preoperative chest x‐ray and chest CT revealed marked tracheal dilation that had been initially overlooked (Figure 1).

Given the persistent air leak, we adapted an approach typically used for tracheoesophageal fistula (TEF) management: the ETT was advanced past the carina into the right main bronchus, then gradually withdrawn until bilateral breath sounds were audible, and securely positioned (modified TEF‐blind method). Although adequate minute ventilation was achieved, damp gauze packing was placed around the oropharynx to further minimize gas leakage. Anaesthesia was maintained with propofol in an oxygen‐air mixture, and the procedure was completed without complications. Postoperatively, the same management strategy was employed in the intensive care unit (ICU), resulting in no symptoms of aspiration or tracheal injury, and the patient was successfully extubated the following day.

## 3 Discussion

Tracheobronchomegaly (MKS) is a rare syndrome characterised by pathological dilation of the trachea, exceeding 21 mm in women and 25 mm in men, along with dilation of the main bronchi on inspiration. The maximum diameter of fully inflated cuffs of an 8‐mm ID tube is up to 25–28 mm while the maximum transverse diameter of the trachea measured 33.29 mm as observed on the CT scan. This anatomical abnormality resulted in inadequate cuff seal despite using progressively larger ETTs. The causes of tracheobronchomegaly can be congenital, such as connective tissue disorders, or acquired, often resulting from conditions that lead to atrophy of the smooth muscle in the tracheobronchial tree, which may be due to factors like compromised blood flow or infections. This syndrome's clinical appearance ranges from asymptomatic to recurrent respiratory infections caused by inadequate mucus clearance and debris. Patients frequently have chronic coughs, shortness of breath, and bronchiectasis. This syndrome can *be difficult* to detect before general anaesthesia, particularly because it may be asymptomatic or because airway stenosis may take precedence over other observable signs on a chest x‐ray (CXR). Indeed, MKS represent the significant anaesthetic challenge due to the risk of significant air leakage around the tube during mechanical ventilation and the potential for pulmonary aspiration.

The management of airway leaks in MKS requires a systematic approach. Our modified approach, adapting principles from tracheoesophageal fistula management, involved advancing the tube into the right main bronchus followed by pulling back the ETT until heard bilateral breathing sounds and firmly securing it. However, this blind advancement technique carries risks of bronchial injury [1, 2, 3].

Fiberoptic bronchoscopy allows direct visualisation for optimal tube positioning and can confirm proper placement without blind manipulation. Laryngeal mask airways (LMAs) may provide an effective seal in some cases, particularly when endotracheal intubation proves challenging. Recent literature by Cheon et al. emphasises the importance of meticulous cuff pressure management and subglottic positioning in MKS patients, focusing on identifying the narrowest tracheal segment for optimal cuff seal [4].

Oropharyngeal gauze packing represented a desperate measure in a critical situation and carried theoretical risks of aspiration and airway obstruction. This technique likely provides minimal benefit for reducing air leaks around an ETT cuff and should be used with caution.

Our management approach shares similarities with previously reported strategies but demonstrates several distinctive adaptations. Wang et al. explored the use of a laryngeal mask airway combined with a modified double‐lumen Foley catheter for lung isolation. However, this method was impractical in lengthy operations with unpredictable airway stability [5]. Xiong et al. advocated subglottic ETT repositioning, comparable to our approach but without the initial bronchial advancement step that proved crucial in our case [6]. There is no consensus regarding the management of the airway in patients with MKS during general anaesthesia, particularly those undergoing one‐lung isolation procedures. The airway management requires careful consideration and tailored strategies to ensure safety and effectiveness during anaesthesia. MKS should be suspected when there is persistent air leakage from the ETT around the cuff and a difference between the tidal volume setting and the detected volume during mechanical ventilation.

This report has several limitations. First, the single‐case nature limits generalizability of findings. Second, the retrospective diagnosis of MKS highlights institutional gaps in preoperative airway evaluation. Finally, while our technique proved effective, prospective validation in larger cohorts is warranted.

In conclusion, this case underscores the critical importance of preoperative recognition of MKS to anticipate and manage ventilation challenges effectively. The described modified technique, involving bronchial advancement and retraction supplemented by gauze packing, successfully addressed a life‐threatening air leak scenario. However, this approach should be considered a rescue manoeuvre when conventional methods fail. We recommend systematic preoperative tracheal diameter assessment in high‐risk patients and emphasise the value of having advanced airway equipment, including bronchoscopes and larger ETTs, readily available. Anaesthesiologists should be familiar with a hierarchy of airway rescue strategies for managing such difficult cases.

## Author Contributions

A.K.: conceptualization, investigation, supervision, writing‐review and editing, and validation. V.N.B.: conceptualization, writing‐review and editing, and validation. F.K.: conceptualization, investigation, writing – original draft and validation. All the authors agreed to be accountable for all aspects of the work in ensuring that questions related to the accuracy or integrity of any part of the work are appropriately investigated and resolved.

## Funding

The authors have nothing to report.

## Ethics Statement

This study received approval from the Ethics Committee of Shiraz University of Medical Sciences (IR.SUMS.REC.1403.410).

## Consent

Written informed consent was obtained from the patient for publication of this case report and any accompanying images. A copy of the written consent is available for review by the Editor‐in‐Chief of this journal.

## Conflicts of Interest

The authors declare no conflicts of interest.

## Data Availability

More detailed data regarding this manuscript can be accessed upon request from the corresponding author.

## References

[1] R. Akgedik , H. Karamanli , D. Kizilirmak , et al., “Mounier–Kuhn Syndrome (Tracheobronchomegaly): An Analysis of Eleven Cases,” Clinical Respiratory Journal 12, no. 3 (2018): 885–889. 28026118 10.1111/crj.12600

[2] I. I. Ayub and R. Vengadakrishnan , “Diseases, Mounier–Kuhn Syndrome,” Tuberculosis Respiratory Disease 86, no. 1 (2022): 59–60. 10.4046/trd.2022.0123 PMC9816488 36514182

[3] E. Krustins , “Mounier‐Kuhn Syndrome: A Systematic Analysis of 128 Cases Published Within Last 25 Years,” Clinical Respiratory Journal 10, no. 1 (2016): 3–10. 25130790 10.1111/crj.12192

[4] B. Cheon , J. H. Lee , J. H. Kim , and S. M. Hwang , “Airway Management of a Patient With Mounier–Kuhn Syndrome During General Anesthesia—A Case Report,” Anesthesia and Pain Medicine 19, no. 2 (2024): 156–160. 38725171 10.17085/apm.23172 PMC11089299

[5] S. N. Wang , A. S. Wu , J. B. Miao , S. Chen , and J. Jiang , “Airway Management for a Patient With Tracheobronchomegaly Undergoing Lobectomy: A Case Report,” BMC Anesthesiology 23, no. 1 (2023): 357. 37919658 10.1186/s12871-023-02324-5 PMC10621132

[6] J. Xiong , Q. Zhou , Y. Li , Y. Sun , and Y. Zhang , “Unexpected Curious Cause of Serious Air Leakage After Endotracheal Intubation: A Case Report of Tracheobronchomegaly and Literature Review,” Frontiers in Surgery 9 (2022): 961186. 36081585 10.3389/fsurg.2022.961186 PMC9445417

